# Magnetic Nanoparticle Thermometer: An Investigation of Minimum Error Transmission Path and AC Bias Error

**DOI:** 10.3390/s150408624

**Published:** 2015-04-14

**Authors:** Zhongzhou Du, Rijian Su, Wenzhong Liu, Zhixing Huang

**Affiliations:** 1 School of Automation, Huazhong University of Science and Technology, Wuhan 430074, China; E-Mails: duzhongzhou@hust.edu.cn (Z.D.); 13297092710@163.com (Z.H.); 2 School of Computer and Communication Engineering, Zhengzhou University of Light Industry, Zhengzhou 450002, China; E-Mail: zzsrj@126.com; 3 Key Laboratory of Image Processing and Intelligent Control, Huazhong University of Science and Technology, Wuhan 430074, China

**Keywords:** magnetic nanoparticle thermometer (MNPT), error source, error transfer, AC bias, non-invasive temperature measurement, magnetic nanoparticles (MNPs)

## Abstract

The signal transmission module of a magnetic nanoparticle thermometer (MNPT) was established in this study to analyze the error sources introduced during the signal flow in the hardware system. The underlying error sources that significantly affected the precision of the MNPT were determined through mathematical modeling and simulation. A transfer module path with the minimum error in the hardware system was then proposed through the analysis of the variations of the system error caused by the significant error sources when the signal flew through the signal transmission module. In addition, a system parameter, named the signal-to-AC bias ratio (*i.e.*, the ratio between the signal and AC bias), was identified as a direct determinant of the precision of the measured temperature. The temperature error was below 0.1 K when the signal-to-AC bias ratio was higher than 80 dB, and other system errors were not considered. The temperature error was below 0.1 K in the experiments with a commercial magnetic fluid (Sample SOR-10, Ocean Nanotechnology, Springdale, AR, USA) when the hardware system of the MNPT was designed with the aforementioned method.

## 1. Introduction

Non-invasive thermometry is of great significance in industrial and biomedical application research. The MNPT is a novel tool of non-invasive temperature measurement and has the potential of observing heat transfer and control in the micro-scale and biological research fields [1,2,3]. This tool has unparalleled advantages in internal temperature measurement of the integrated circuit (IC), the temperature measurement and control of living cells, cancer hyperthermia and others [4,5,6,7,8,9,10,11,12,13,14]. However, the measured temperatures are expected to have an error below 0.1 K, given the high requirement in the temperature precision in the processes of heat transfer and control in the micro-scale field and the observation of the behavior of cells in biological research. The MNPT, which is currently limited by the measuring precision, is expected to be improved for its applications in the micro-scale and biological fields.

The magnetization curve of MNPs is sensitive to temperature, which is able to be employed for temperature measurement. The nonlinear magnetization response of the MNPs in an AC time-varying magnetic field contains the first, third, fifth and other odd harmonics. Therefore, the amplitudes of the harmonics of the magnetization response of MNPs are substituted for the temperature. Weaver, J.B. studied the harmonic ratio to estimate the temperature of MNPs with an accuracy of 0.3 K and reported that the magnetic spectroscopy of Brownian motion was also used for temperature estimation [15,16,17]. Our group proposed different temperature measurement models and methods of the MNPT in different excitation magnetic fields. As the first harmonic includes abundant temperature information, the first and third harmonic amplitudes were substituted for the temperature in order to improve the precision of MNPT. The first and third harmonic amplitude model are described by the first order Langevin function when the working frequency is very low (<1 kHz), so that the temperature of the MNPs can be estimated [5,18,19,20,21,22,23,24].

Several factors may bring in temperature measurement errors in the hardware system, such as deformation of the mechanical structure, the nonlinear characteristic of electrical parts and the machining error of the mechanical parts. The deformation and machining error of the mechanical structure of the Helmholtz coils result in the deviation of its radius and turns. The nonlinear characteristic of the power amplifier introduces the deviation of the voltage gain and the DC bias of the power amplifier. However, all of the error sources in the hardware system and the variation of the errors as the signal flew through the signal transmission module have not been clearly investigated for the high-precision MNPT. In addition, in theory, the output signal of the differential coils is zero when an AC excitation magnetic field exists. In practice, the output signal of the differential coils is not zero, which is named the AC bias, resulting from the asymmetric differential coils. The AC bias has not been studied for how it influences the precision of the MNPT. It is important to study the factors mentioned above for improving the precision of the MNPT.

The present study focuses on two aspects: the errors in the hardware system and the AC bias. For the aspect of the errors in the hardware system, the paper aims to establish a signal model in the hardware system, to determine the error sources that significantly affect measurement precision, to analyze the relation between the errors in each part of the hardware system and the temperature error, to reduce the hardware system error by adjusting the sign and value of the each error source according to the variation of the significant influence of error and to improve the measurement precision of the MNPT. For the aspect of the AC bias, the AC bias is also identified as a major influence on the measurement precision of the MNPT. The minimum signal-to-AC bias ratio was proposed for ensuring that the temperature error was below 0.1 K without considering the other errors.

## 2. System Constitution and Temperature Solution

**Figure 1:**
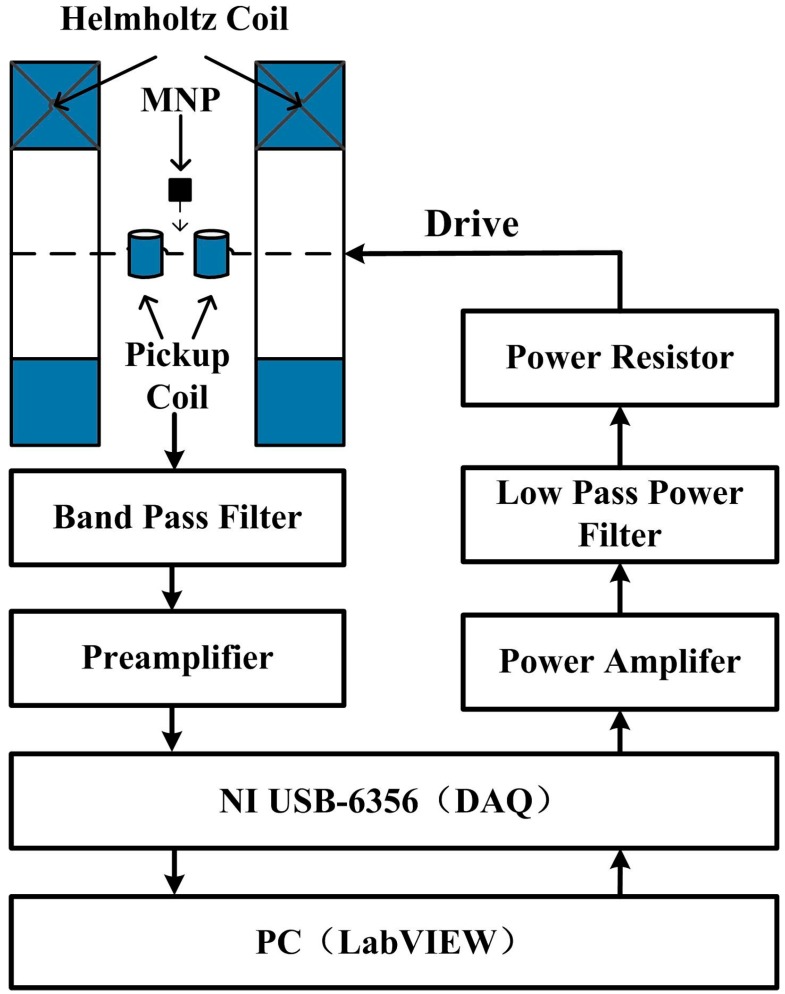
System structure of the MNPT. DAQ, data acquisition card.

The MNPT can be divided into three parts (as shown in Figure 1): the excitation module for generating the AC magnetic field, the detecting module for measuring the odd harmonics of the magnetization of MNPs and the software for signal post-processing and solving the temperature.

The signal source of the AC exciting magnetic field was first output using a data acquisition card (DAQ, NI-USB-6356, National Instruments Corporation, Austin, TX, USA) and then amplified by a power amplifier (AE-7224, AE Techron Inc., Elkhart, IN, USA) and filtered through a ninth-order elliptic low-pass filter (self designed), after which it was finally transmitted to the Helmholtz coils to generate the AC exciting magnetic field. The sampling resistor, which is a power resistor installed in series to the load circuit for monitoring the excitation current of the Helmholtz coils and the negative feedback control algorithm, was used to stabilize the exciting magnetic field, the fluctuation of which was less than 0.01%.

A pair of differential hollow coils was used to probe the weak magnetization of the MNPs. The chosen signals were filtered through a band-pass filter and were then amplified using a preamplifier (Stanford preamplifier, SR560, Sunnyvale, CA, USA), sampled by the DAQ (NI-USB-6356). All of the sample data were processed in PC-LabVIEW. The digital phase-sensitive detection algorithm (DPSD) was used to extract the amplitudes of the first and the third harmonics of the magnetization of the MNPs.

For magnetic nanoparticles (MNPs), the relaxation phenomena happen when an excitation magnetic field is applied. The relaxation phenomena are relative to the excitation field and the particle size. Generally, the MNPs with a small particle size and narrow distribution of the particle size are used to avoid the relaxation effects when the working frequency of the MNPT is low. According to the results reported by Frank Ludwig, the relaxation effects on the MNPT are negligible when the average particle size is smaller than 30nm, the particle size distribution is narrow and the excitation field has frequencies lower than 1 kHz [25,26]. As such, the first-order Langevin function describing the superparamagnetism of the MNPs is specified by [19,20,21,22,23,24]:

(1) $$M=\phi M_{s}\left(\coth\left(\frac{M_{s}VH}{k_{B}T}\right)-\frac{k_{B}T}{M_{s}VH}\right)$$

where ϕ is the volume fraction of MNPs, *M_s_* is the saturation magnetization, *V* is the particle’s volume, *k_B_* is the Boltzmann constant, *T* is the absolute temperature and *H* is the external excitation magnetic field.

When the excitation field is set to be a sinusoid waveform with a single frequency, which is expressed as *H = H_0_sin*(ω*t*) with ω = 2π*f*, Equation (1) can be transformed through Taylor expansion:

(2) $$M=A_{1}\sin\left(\omega t\right)+A_{3}\sin\left(3\omega t\right)+A_{5}\left(5\omega t\right)+...$$

where *A_1_* and *A_3_* are the first and third harmonic amplitudes of MNP magnetization, respectively. *A_1_* and *A_3_* include the temperature information of MNPs. Therefore, the temperature-measuring principle of the MNPT is the establishment of the harmonic amplitude-temperature equation through the amplitude of the first and the third harmonics, which are the odd harmonics of the magnetization of MNPs under a single-frequency exciting magnetic field [19,25,27]. The harmonic amplitude-temperature equation is as follows.

(3) $$\left\{ \begin{array}{l} A_{1}\text{=}\frac{xy}{3}\text{-}\frac{xy^{3}}{60}\text{+}\frac{xy^{5}}{756}\text{-}\frac{xy^{7}}{8640}+\frac{xy^{9}}{95040}\\ A_{3}\text{=}\frac{xy^{3}}{180}\text{-}\frac{xy^{5}}{1512}\text{+}\frac{xy^{7}}{14400}-\frac{xy^{9}}{142560} \end{array}\right.$$

where *x = ϕM_s_* and *y = M_s_VH/k_B_/T*.

Equation (3) can be described as follows:

(4) $$\left\{ \begin{array}{l} A_{1}=F(\phi ,T)\\ A_{3}=G(\phi ,T) \end{array}\right.$$

Solving Equation (4) allows temperature measurement by using the Levenberg–Marquardt algorithm (L–M) [20,21,22,23,24].

## 3. Model and Method

**Figure 2:**
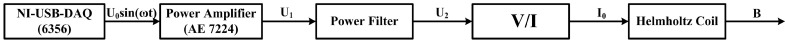
Signal transmission path of the magnetic field excitation module.

According to the signal transmission path, the hardware part of the MNPT is also divided into two parts: the magnetic field excitation module and the magnetic detection module. The signal transmission in the magnetic field excitation module is shown in Figure 2. The power amplifier, power filter and Helmholtz coils (electromagnetic conversion part) were constructed to generate the excitation signals. The DAQ output was assumed to be an ideal sinusoidal signal *U_0_sin*(ω*t*) with the angular frequency ω = 2π*f*. An additional phase and a DC-biased voltage were added as the signal passed through the power amplifier. The output signal of the power amplifier is described as:

(5) $$U_{1}=k_{1}U_{0}\sin\left(\omega t+\theta_{1}\right)+c_{1}$$

where *k*_1_ is the voltage gain of the power amplifier, *c_1_* is the DC bias of the power amplifier and θ_1_ is the phase angle of the power amplifier. Extra errors were caused as the signal passed through the power filter:

(6) $$U_{2}=k_{1}k_{2}U_{0}\sin\left(\omega t+\theta_{1}+\theta_{2}\right)+k_{2}c_{1}$$

where *k*_2_ is the attenuation coefficient of the filter within the pass band andθ_2_ is the phase angle of the filter within the band. The filtered voltage signal was then input into the Helmholtz coil, and transformed to the driving current in the V/I module (voltage-current converter) according to Ohm’s Law [28,29], which is expressed as:

(7) $$I=\frac{U_{2}}{Z}=\frac{k_{1}k_{2}U_{0}}{\sqrt{R_{1}^{2}+\omega^{2}L^{2}}}\sin\left(\omega t+\theta_{1}+\theta_{2}-\arctan\frac{\omega L}{R_{1}}\right)+\frac{k_{2}c_{1}}{R_{1}}$$

where *R*_1_ is the sum of the power resistance and DC resistance of the Helmholtz coils and *L* is the inductance of the Helmholtz coils. According to the Biot–Savart Law [30,31], the final excitation magnetic field at the geometrical center of the Helmholtz coils is modeled as:

(8) $$\begin{array}{ll} B & ={\left(\frac{4}{5}\right)}^{3/2}\frac{\mu_{0}N_{0}I}{r_{0}}={\left(\frac{4}{5}\right)}^{3/2}\frac{\mu_{0}N_{0}}{r_{0}}\left\{\frac{k_{1}k_{2}U_{0}}{\sqrt{R_{1}^{2}+\omega^{2}L^{2}}}\sin\left(\omega t+\theta_{1}+\theta_{2}-\arctan\frac{\omega L}{R_{1}}\right)+\frac{k_{2}c_{1}}{R_{1}}\right\}\\ \; & =g_{1}\left(N_{0},r_{0},U_{0},R_{1},k_{1},k_{2},\theta_{1},\theta_{2},L,c_{1}\right) \end{array}$$

where *r_0_* is the radius of the Helmholtz coils and *N_0_* is the number of turns of the Helmholtz coils.

**Figure 3:**
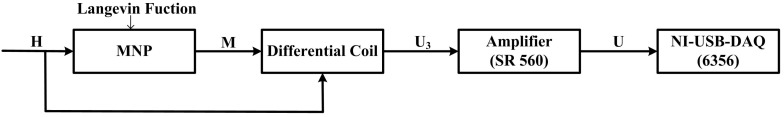
Signal transmission path of the magnetic detection module.

When the excitation magnetic field was applied, the magnetization of MNPs could be described by Equation (1). In order to analyze this easily, the errors introduced by Equation (1) were neglected. The responding signal is then detected and transferred to the signal transmission path of the magnetic detection module, as shown in Figure 3. According to the signal transmission direction, the detection module is constructed using the differential coil, amplifier (Stanford preamplifier, SR560) and data acquisition device (NI-USB-6536).

According to Faraday’s law of induction coils [32,33], the voltage induced in the closed turns of a coil is proportional to the time rate of change of the flux linked with the coil. The output signal of the differential coil [34] is expressed as:

(9) $$\begin{array}{ll} U_{3} & =-n_{1}\frac{d\phi_{1}}{dt}-\left(-n_{2}\frac{d\phi_{2}}{dt}\right)\\ \; & =-n_{1}s_{1}\mu_{0}\frac{d\left(H+M\right)}{dt}+n_{2}s_{2}\mu_{0}\frac{d\left(H-M\right)}{dt}\\ \; & =\left(-n_{1}s_{1}+n_{2}s_{2}\right)\mu_{0}\frac{dH}{dt}-\left(n_{1}s_{1}+n_{2}s_{2}\right)\mu_{0}\frac{dM}{dt} \end{array}$$

where *n*_1_ is the turn of Coil 1, *s*_1_ is the effective cross-sectional area of Coil 1, *n*_2_ is the turn of Coil 2 and *s*_2_ is the effective cross-sectional area of Coil 2. After being amplified by the preamplifier, the output voltage is:

(10) $$U=k_{3}U_{3}+c_{2}$$

where *k*_3_ is the voltage gain of the pre-amplifier and *c_2_* is the DC bias of the preamplifier.

By summarizing Equations (9) and (10), the final signal output from the hardware system can be integrated as follows:

(11) $$U=\left\{ \begin{array}{l} g_{2}\left(n_{1},s_{1},k_{3},c_{2},M\right)\;where\;n_{1}=n_{2},s_{1}=s_{2}\\ g_{2}\left(n_{1},n_{2},s_{1},s_{2},k_{3},c_{2},H,M\right)\;others \end{array}\right.$$

When the MNPs were ideal samples, Equation (11) can be transformed to Equation (12) according to Equation (1).

(12) $$U=\left\{ \begin{array}{l} g_{3}\left(n_{1},s_{1},k_{3},c_{2},H\right)\;where\;n_{1}=n_{2},s_{1}=s_{2}\\ g_{3}\left(n_{1},n_{2},s_{1},s_{2},k_{3},c_{2},H\right)\;others \end{array}\right.$$

By summarizing Equations (8) and (12),

(13) $$U=\left\{ \begin{array}{l} g\left(n_{1},s_{1},k_{1},k_{2},k_{3},\theta_{1},\theta_{2},c_{1},c_{2},U_{0},N_{0},r_{0},R_{1},L\right)\;where\;n_{1}=n_{2},s_{1}=s_{2}\\ g\left(n_{1},n_{2},s_{1},s_{2},k_{1},k_{2},k_{3},\theta_{1},\theta_{2},c_{1},c_{2},U_{0},N_{0},r_{0},R_{1},L\right)\;others \end{array}\right.$$

In terms of the error transfer theory [35,36,37,38], the error of the whole hardware system introduced in the MNPT can be modeled by:

(14) $$\Delta U=\left\{ \begin{array}{l} \frac{\partial g}{\partial n_{1}}\Delta n_{1}+\frac{\partial g}{\partial s_{1}}\Delta s_{1}+\frac{\partial g}{\partial k_{1}}\Delta k_{1}+\frac{\partial g}{\partial k_{2}}\Delta k_{2}+\frac{\partial g}{\partial k_{3}}\Delta k_{3}+\frac{\partial g}{\partial \theta_{1}}\Delta \theta_{1}\\ +\frac{\partial g}{\partial \theta_{2}}\Delta \theta_{2}+\frac{\partial g}{\partial c_{1}}\Delta c_{1}+\frac{\partial g}{\partial c_{2}}\Delta c_{2}+\frac{\partial g}{\partial U_{0}}\Delta U_{0}+\frac{\partial g}{\partial N_{0}}\Delta N_{0}\\ \;+\frac{\partial g}{\partial r_{0}}\Delta r_{0}\;+\frac{\partial g}{\partial R_{1}}\Delta R_{1}+\frac{\partial g}{\partial L}\Delta L\;where\;n_{1}=n_{2},s_{1}=s_{2}\\ \;\\ \frac{\partial g}{\partial n_{1}}\Delta n_{1}+\frac{\partial g}{\partial n_{2}}\Delta n_{2}+\frac{\partial g}{\partial s_{1}}\Delta s_{1}+\frac{\partial g}{\partial s_{2}}\Delta s_{2}+\frac{\partial g}{\partial k_{1}}\Delta k_{1}+\frac{\partial g}{\partial k_{2}}\Delta k_{2}+\frac{\partial g}{\partial k_{3}}\Delta k_{3}\\ +\frac{\partial g}{\partial \theta_{1}}\Delta \theta_{1}\;+\frac{\partial g}{\partial \theta_{2}}\Delta \theta_{2}+\frac{\partial g}{\partial c_{1}}\Delta c_{1}+\frac{\partial g}{\partial c_{2}}\Delta c_{2}+\frac{\partial g}{\partial U_{0}}\Delta U_{0}+\frac{\partial g}{\partial N_{0}}\Delta N_{0}\\ \;+\frac{\partial g}{\partial r_{0}}\Delta r_{0}\;+\frac{\partial g}{\partial R_{1}}\Delta R_{1}+\frac{\partial g}{\partial L}\Delta L\;others \end{array}\right.$$

The mathematical model of the error in the hardware system can be obtained by placing each parameter of the hardware system into Equation (14). As the 1st harmonic amplitudes (*A*_1_) and the 3rd harmonic amplitudes (*A*_3_) of the MNPs magnetization were obtained from the output signal *U* in the hardware system using the DPSD, the temperature of MNPs was obtained from the *A*_1_ and *A*_3_ according to Equation (4); the temperature measurement error of the MNPT was influenced by the deviation of the output signal of the hardware system (*∆U*). To analyze easily, the system errors introduced by the DPSD and L-M algorithm were neglected.

Each part in Equation (14) theoretically leads to temperature errors of the MNPT. Thus, the errors in the hardware system were mainly caused by the following factors: the signal source *U*_0_, the number of turns of detection coil *n*_1_ and *n*_2_, the cross-sectional area of detection coils *s*_1_ and *s*_2_,the voltage gain of power amplifier *k*_1_, the phase shift of power amplifier θ_1_, the attenuation coefficient of power filter within the pass band *k*_2_, the phase shift of power filter θ_2_, the radius of the Helmholtz coils *r*_0_, the number of the turns of the Helmholtz coils *N*_0_, the inductance of the Helmholtz coils *L*, the DC resistance of the Helmholtz coils *R*_1_, the voltage gain of the preamplifier *k*_3_ and the DC bias of the preamplifier *c*_1_.

The simulations of the temperature error with the variation of the error sources were designed when *n*_1_* = n*_2_ and *s*_1_* = s*_2_, that is there is no AC bias in the hardware system. The temperature error resulted from the deviation of *A*_1_ and *A*_3_ according to Equation (3), which are introduced by the deviation of the output signal of the hardware system in the process of the harmonic amplitudes (*A*_1_, *A*_3_) obtained by the DPSD. Therefore, the simulations were designed to analyze how each significant factor mentioned above influences the deviation of *A*_1_, *A*_3_ and the temperature error. Based on the method described, the significant effect factors and then the deviation range of each significant effect factor were determined, which serves as the reference for our design of the hardware system’s parameters.

Note that the deviation of the significant effect error may be positive or negative. According to the signal transmission path, the fluctuation rule of the significant effect error in the process of signal transmission can be described clearly. The error of the output signal (*∆r*) in the hardware system resulting from significant effect error changes with the sign and value of the interaction among the significant effect errors. Therefore, the minimum error transmission path can be determined by matching the sign and value of the significant effect errors to each other. The hardware system with the minimum error can be designed through this method.

The preceding analysis of both the individual error source and the error transfer path is based on the assumption that the geometric structure and the electrical character of the differential coils are completely consistent with each other. As such, the output signal of the differential coils is zero when an AC excitation magnetic field exists. In practice, an AC bias exists in the output signal because the two detection coils are asymmetric. We consider the AC bias to be a kind of noise in the output signal, so that the signal-to-AC bias ratio is simulated for the observation of the measurement error. The AC bias in the hardware system, which is an important significant effect error, is considered in the simulation. The higher the AC bias, the lower the signal strength and the higher the temperature error. The ratios with the values of 40, 60, 80 and 100 dB are separately considered in the calculation of the temperature. Without considering other errors, the signal-to-AC bias ratio in the hardware system is proposed for below 0.1 K, the temperature error in the simulation.

## 4. Simulation and Experiments

The simulation is firstly applied on the investigation of the significant error sources. Each parameter with five deviations (0.01%, 0.05%, 0.1%, 0.5% and 1%) is used as input in the hardware system for comparison. The errors of the first and third harmonics, their ratio and the final measured temperature are used as the evaluation parameters. The error analysis is then focused on the simulation of the signal transfer path under the influence of the significant error sources to find the signal transfer path with minimal errors. Experiments are implemented for the validation of the conclusions made in simulations. In addition, when the AC bias is considered, simulations on the reaction of AC bias under different SNR values are firstly performed. The minimal SNR in AC bias is proposed when the temperature error was below 0.1 K. Experiments are also implemented for the validation of this conclusion. The MNP-based sample used in this study was a commercial magnetic fluid (Sample SOR-10, Fe_3_O_4_) produced by Ocean Nanotechnology Ltd. Corp. (Springdale, AR, USA), consisting of magnetite nanoparticles with an average diameter of approximately 10 nm. The nanoparticles were surface-coated with oleic acid and dispersed in an organic solvent at a concentration of 25 mg Fe/mL.

### 4.1. Individual Error Source in the Hardware System

**Figure 4:**
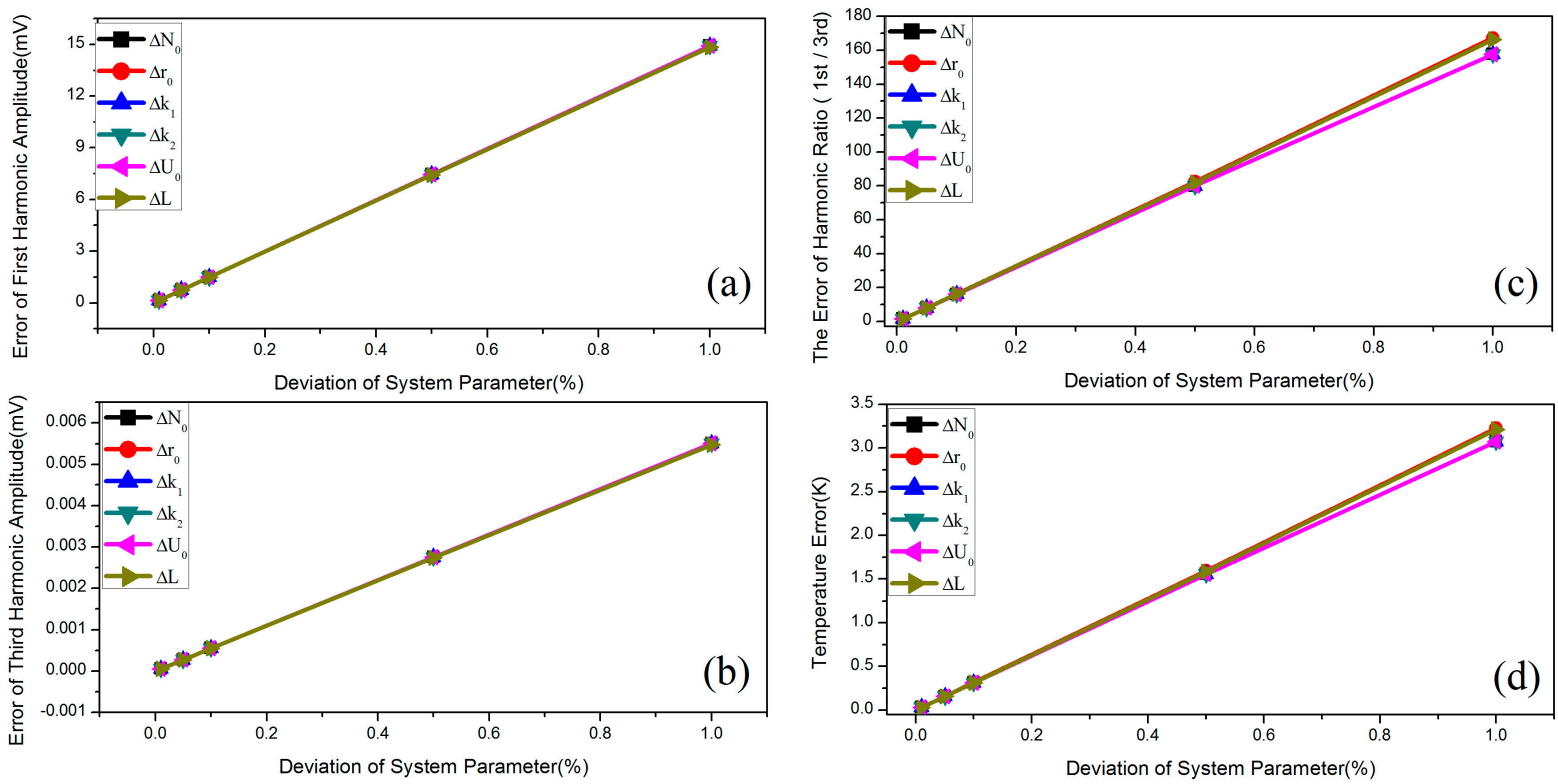
Harmonic measurement error resulting from the deviation of the parameters, such as the radius of the Helmholtz coils (*r*_0_ = 0.1025 m), the turn of the Helmholtz coils (*N*_0_* =* 168), the AC impedance of the Helmholtz coils (*ωL* = 44.06247 Ω), the voltage gain of the power amplifier (*k*_1_ = 7.8), the attenuation coefficient of the power amplifier within the pass band (*k*_2_ = 1) and the signal source (*U*_0_ = 5.75 V). The deviation rates of these parameters are 0.01%, 0.05%, 0.1%, 0.5% and 1%, respectively. The MNPs follow a single particle distribution (10 nm); the saturation magnetic moment is *MsV* = 2.4976 × 10^−19^ emu; the frequency is 375 Hz; the system has no noise and no AC bias. (**a**) represents the error of first harmonic amplitude resulting from the deviation of the parameters; (**b**) represents the error of third harmonic amplitude resulting from the deviation of the parameters; (**c**) represents the error of harmonic ratio (1st/3rd) resulting from the deviation of the parameters; (**d**) represents the temperature error resulting from the deviation of the parameters.

The errors of the first and third harmonics significantly increased with the deviation of some of the error sources (0.01%, 0.05%, 0.1%, 0.5%, 0.1%); as shown in Figure 4a,b, these error sources consist of the radius of the Helmholtz coils *r_0_*,the turn of Helmholtz coils *N_0_*, the inductance of Helmholtz coils *L*, the voltage gain of power amplifier *k*_1_, the attenuation coefficient of power amplifier within the pass band *k*_2_ and the signal source *U*_0_. The error of the ratio between the first and third harmonics follows the same increment as shown in Figure 4c. The temperature error follows the same increment, as presented in Figure 4d. The error of the measured temperature is approximately 0.03 K when the deviation of each error is maintained at 0.01%. When the deviation is increased from 0.01% to 0.1%, the error of the measured temperature is approximately 0.3 K. However, the error increases to 3 K as the error of each source increases to 1%. Thus, each of the parameters mentioned should be precisely adjusted to reduce the measurement error of the MNPT. The simulation results reveal that the temperature error with 0.1 K in the MNPT is able to be reached if the errors of the above parameters are lower than 0.01% without the consideration of the other factors.

**Figure 5:**
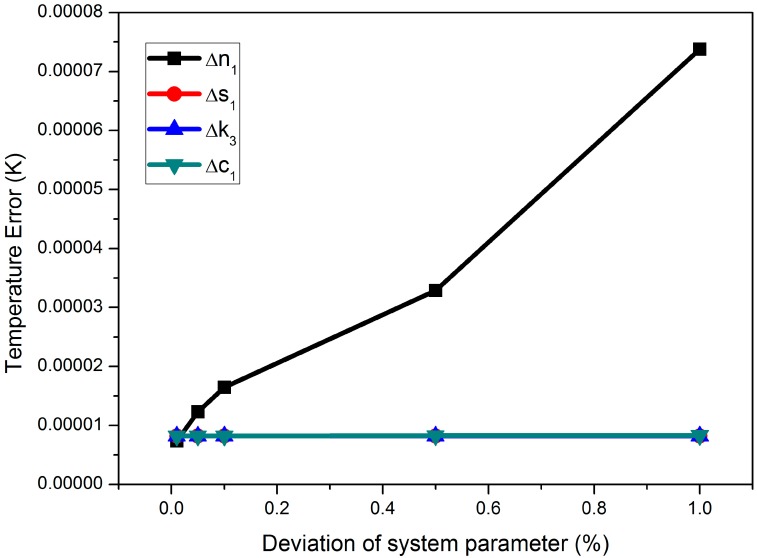
Temperature measurement error resulting from parameters, such as the turns of the detection coil (*n*_1_ = 884, *n_2_* = 884), the cross-sectional area of the detection coil (*s*_1_ = 58.99 mm^2^, *s*_2_ = 58.99 mm^2^), the voltage gain of the preamplifier (*k*_3_ = 1000) and the DC bias of the preamplifier (*c*_1_ = 8.2 mV). The deviation rates of the parameters are 0.01%, 0.05%, 0.1%, 0.5% and 1%, respectively. The mean particle diameter is 10 nm; the saturation magnetic moment is *MsV* = 2.4976 × 10^−19^ emu; the magnetic excitation frequency is 375 Hz; the system has no noise and no AC bias.

Other error sources that have been cataloged as factors with a weak influence on the measurement precision are analyzed in Figure 5. These parameters include the number of turns of the detection coils *n*_1_, the cross-sectional area of the detection coil *s*_1_, the voltage gain of the preamplifier *k*_3_ and the DC bias of the preamplifier *c*_1_.

### 4.2. Error Transfer Path

**Figure 6:**
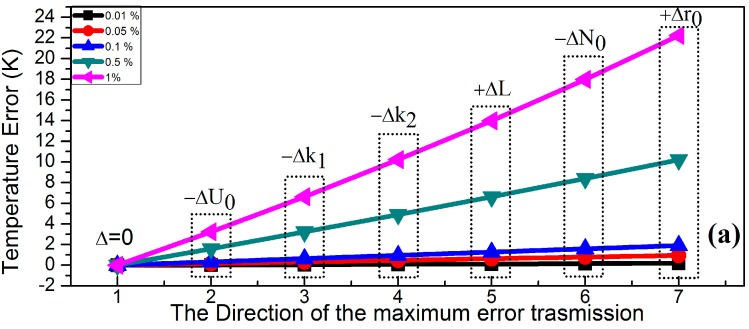

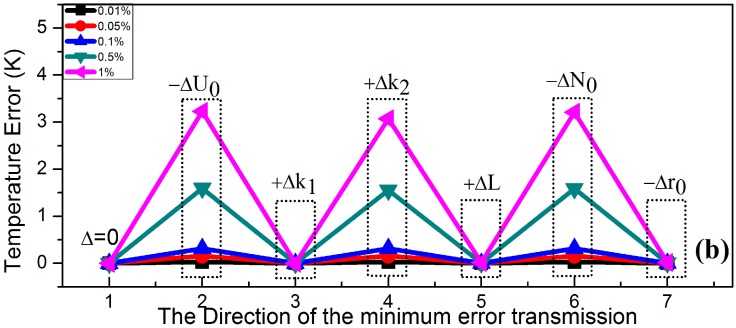
Maximum and minimum transmission direction of the temperature error caused by some factors, such as the radius of the Helmholtz coils (*r*_0_* =* 0.1025 m), the turn of the Helmholtz coils (*N*_0_ = 168), the AC impedance of the Helmholtz coils (*ω* = 44.06247Ω), the voltage gain of the power amplifier (*k*_1_ = 7.8), the attenuation coefficient of the power amplifier within the pass band (*k*_2_ = 1) and the signal source (*U*_0_ = 5.75 V). The deviation of each factor respectively is 0.01%, 0.05%, 0.1%, 0.5%, 1%. (**a**) represents the maximum transmission direction of the temperature error; (**b**) represents the minimum transmission direction of the temperature error.

The error of the measured temperature is also affected by the signal transmission path aside from the errors introduced by individual error sources. The temperature error changes with different error transfer paths. The variations of the individual error sources with 0.01%, 0.05%, 0.1%, 0.5% and 1%, respectively, are simulated in the study of the signal transfer path with maximum and minimum errors. The results are shown in Figure 6. When the error transfer path is set to be the path shown in Figure 6a, which is the maximum error transfer path, the system errors gradually increase as the errors of individual sources increase. Conversely, when the error transfer path in Figure 6b is chosen, that is the minimum error transfer path, the system errors can reach the minimum value as the errors of individual sources counteract with each other.

The temperature error introduced by each effect error reaches the minimum value when the sign of the error from each source in the MNPT is adjusted. The individual error factor with 1% variation is analyzed as an example. When the direction of the individual error with 1% variation follows, as shown in Figure 6a, the accumulated system errors lead to a temperature error of 22 K, which is the biggest error in the transmission module. When the direction of the individual error with 1% variation (*i.e.*, optimal error transmission path) follows, as shown in Figure 6b, the temperature error reaches approximately 0 K because of the mutually compensated errors from each error source. The temperature error is 3.2 K when only −1% error is introduced in the DAQ analog output Δ*U_0_* in the hardware system. However, the temperature error decreases to approximately 0 K when 1% error is simultaneously introduced in the power amplifier Δ*k*_1_. The reason is that the −1% error in Δ*U*_0_ is offset by the 1% error in Δ*k*_1_. The other errors are also analyzed following the method mentioned. Similarly, the errors in Δ*k*_2_, Δ*L*, Δ*N*_0_ and Δ*r*_0_ are mutually diminished. When the smallest error transfer path mentioned is chosen, the temperature error of the MNPT decreases despite the accumulation of errors from different sources in the hardware system. The simulation results of the significant error factors with 0.01%, 0.05%, 0.1% and 0.5% variations are the same with the results of the factors with 1% variation. Therefore, the conclusion is that the temperature precision of the MNPT is able to reach the requirement if the errors in the hardware system compensate for each other, even the individual error sources significantly influence the measurement precision. An important design principle of the hardware system with high precision is thus established. The error sources should follow the value given in Equation (13) if the temperature error is affected by the minimal error in the hardware system, where δ is the deviation of the individual error source.

(15) $$\left\{ \begin{array}{l} 0<\Delta k_{1}<\delta \\ 0<\Delta L<\delta \\ -\delta <\Delta r_{0}<0\\ 0<\Delta k_{2}<\delta \\ -\delta <\Delta N_{0}<0 \end{array}\right.$$

**Figure 7:**
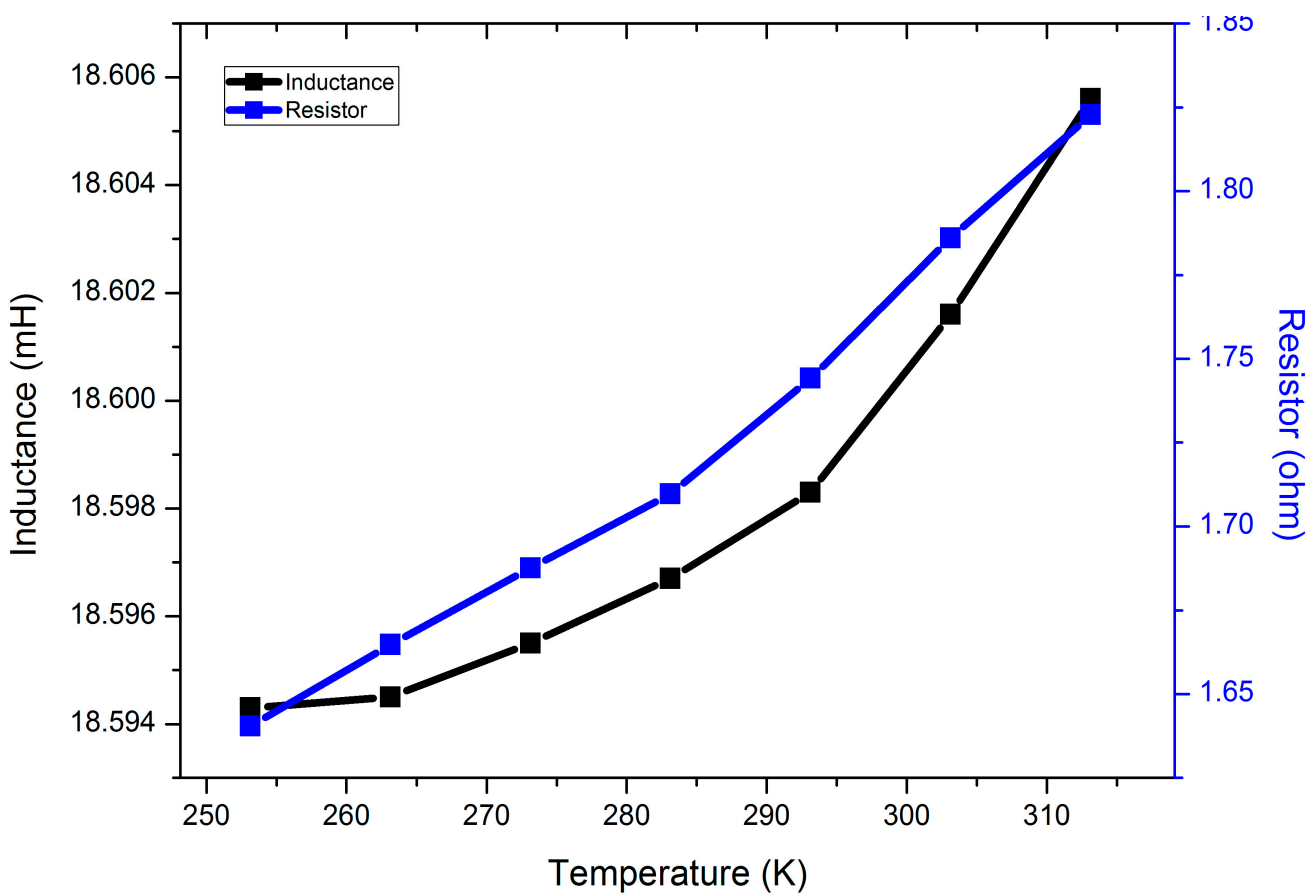
The inductance and resistance of Helmholtz coils varied with temperature. The temperature range is from 253 K to 313 K.

There are mainly four significant error factors in the hardware system: power amplifier, power filter, NI DAQ and the Helmholtz coils. The deviations of the voltage gain of the power amplifier may be offset by adjusting the output of the NI DAQ according to the minimum transmission path. The deviation of the attenuation coefficient of the power filter within the pass band may be offset by adjusting the parameter of the power filter. As such, the Helmholtz coils are found to be the most significant factor in the hardware system. The experiments on the Helmholtz coils in different temperatures are designed for the analysis of the influence of the coils on the MNPT. The Helmholtz coils are put into the temperature chambers. The values of the resistance, the inductance and the capacitance of the Helmholtz coils are measured at different temperatures by using the impedance analyzer (4294A, Agilent Technology, Santa Clara, CA, USA). The experiment temperatures are set to be 253 K to 313 K with a step size of 10 K. The values of the resistance, the inductance and the capacitance increase as the temperature rises, as shown in Figure 7, which indicates the change of the size, length and the number of turns of the coils. The trend of the size, length and the number of turns of the coils only follow the maximum error transmission path according to Figure 6a.

**Figure 8:**
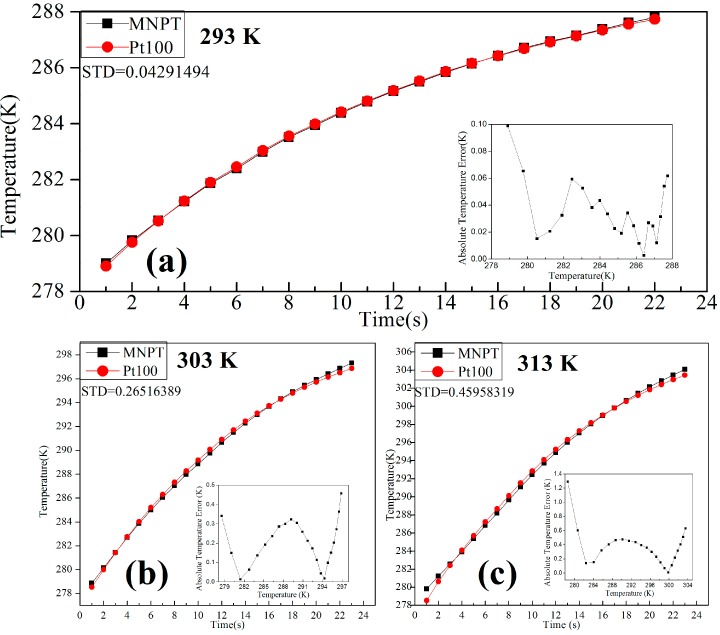
The error of the MNPT under different temperatures for the chambers. (**a**–**c**) represent different measurement error of the MNPT with respect to the temperatures for the chamber of 293 K, 303 K and 313 K.

In order to validate the simulation results in Figure 6a, experiments as follows were designed. Firstly, the MNPT was put into an air-conditioned room, for which the temperature was kept at 293 K. Secondly, each part of the hardware system was adjusted following the minimum error transmission path in Figure 6b. The magnetic nanoparticle samples were cooled to 275 K in an ice water bath. The samples at 275 K were then exposed to air for natural heating in the air-conditioned room. The MNPT and Pt100 thermometer (calibrated by 5627A, Fluke Corporation, Everett, WA, USA) were simultaneously used for real-time temperature measurement in the heating process. The temperature of the samples gradually rose from 275 K to 288 K. The temperature measured by both the MNPT and the Pt100 were recorded as shown in Figure 8a. The red curve is the rising temperature curve measured by the Pt100 temperature sensor. The black curve is the rising temperature curve measured by the MNPT. The changes in the temperature measured by the two methods were almost the same. The measurement error between the two methods was less than 0.1 K in the range of 275 K to 288 K, as shown in the subgraph in Figure 8a. Thirdly, the Helmholtz coils were put into the temperature chambers, for which the temperature was fixed at 303 K and 313 K, respectively. The aforementioned experiment was repeated. The experiment results are shown in Figure 8b,c. The temperature measurement error of the MNPT increases with the temperature of the chambers. The reason is that the error is transmitted along the maximum error transmission path. It is suggested to soak the Helmholtz coils in silicone oil using the circulation cooling system for a stabilized temperature environment.

### 4.3. Error from the AC Bias in the System

**Figure 9:**
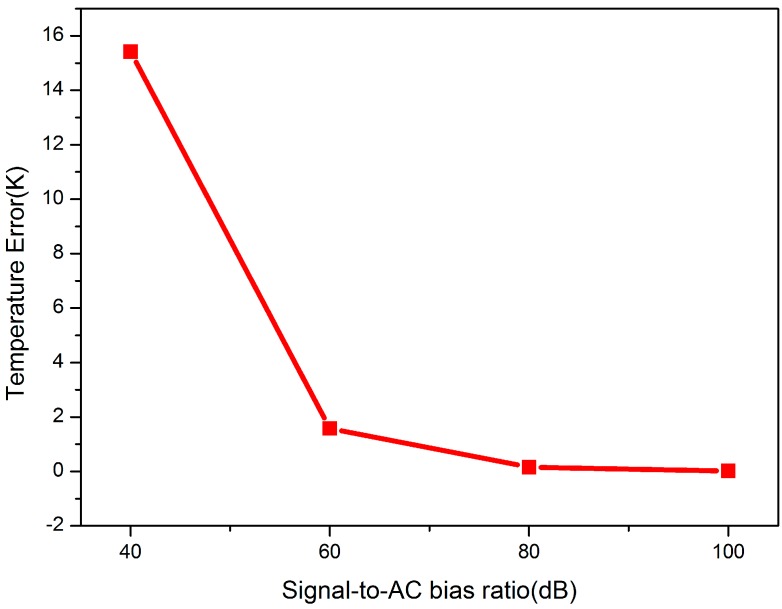
Temperature error caused by the AC bias under different signal-to-AC bias ratios (40, 60, 80, 100 dB).

The precision of the measured temperature fluctuated significantly along with variation in the AC bias in the system, as shown in Figure 9. When the signal-to-AC bias ratio was 40 dB, which meant that a remarkable AC bias effect emerged in the system, the error of the measured temperature was quite large, which exceeded 15 K. The error decreased rapidly along with an improvement in the signal-to-AC bias ratio from 40 dB to 60 dB. When the signal-to-AC bias ratio reached 100 dB, which meant that the AC bias was inconspicuous, the error decreased to 0.0158 K. Hence, the elimination of the AC bias was a necessary step to maintain the error of the measured temperature below 0.1 K. The signal-to-AC bias ratio is recommended to be larger than 80 dB, as shown in Figure 9.

**Figure 10:**
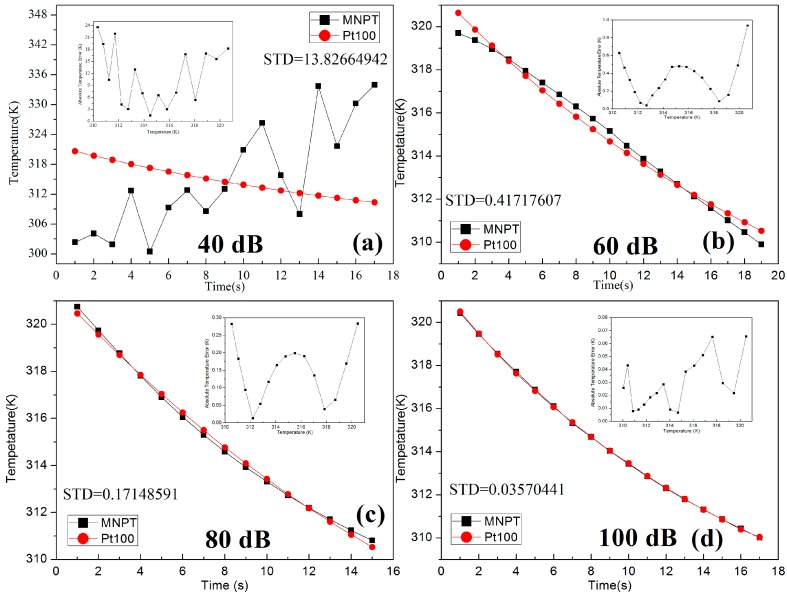
The measurement error of the MNPT resulted from the different signal-to-AC bias ratio. (**a**–**d**) represent different measurement error with respect to the signal-to-AC bias ratio of 40 dB, 60 dB, 80 dB and 100 dB.

Experiments with different signal-to-AC bias ratios (40 dB, 60 dB, 80 dB and 100 dB) are implemented in the MNPT for the validation of the simulation. Experiments are all finished in the air-conditioned room with a constant temperature of 298 K. The experimental procedures were as follows. First, the magnetic nanoparticle samples were heated to 325 K in a water bath. The samples at 325 K were then exposed to air for natural cooling in the air-conditioned room. The MNPT and Pt100 thermometer were simultaneously used for real-time temperature measurement in the cooling process. The temperatures of the samples gradually decreased from 321 K to 310 K within 1 min. The temperatures measured by both the MNPT and the Pt100 were recorded as shown in Figure 10. The red curve is the temperature drop curve measured by the Pt100. The black curve is the temperature drop curve measured by the MNPT. When the signal-to-AC bias ratio is 100 dB, the changes in the temperature measured by the two methods were almost the same. The measurement error between the two methods was less than 0.1 K in the range of 321 K to 310 K, as shown in the subgraph in Figure 10d. The results of the cooling experiments under different signal-to-AC bias ratios are shown in Figure 10. As the signal-to-AC bias ratio decreases, the errors of the measured temperature increase quickly. The conclusions in the simulation are validated in the experiments.

## 5. Discussion

In the design of the Helmholtz coils, materials that maintain a stable structure should be used for the mechanical structure of the Helmholtz coils. The machining process technology needs to be controlled precisely, and a working environment with a constant temperature should be provided for the Helmholtz coils. It is suggested that the Helmholtz coils be soaked in silicone oil using the circulation cooling system to maintain a stable temperature. The machining process technology needs to be also controlled precisely. The parameters were measured by the impedance analyzer (4294A, Agilent Technology) in the air-conditioned room (293 K) after the Helmholtz coils were settled down. When the deviation of the error source in the Helmholtz coils did not follow Equation (15), the parameters of the Helmholtz coils were offset by adding extra resistors and inductors. The error source caused by the Helmholtz coils can be cancelled through the calibration procedures mentioned above. For an easier analysis, some factors influence the errors of the measured temperature in the MNPT. However, these factors, such as the truncation error brought into the discretization of the Langevin function, the DPSD, the L-M algorithm, the particle sizes, the particle distributions, and so on, result in measurement errors, as well. The temperature precision of the MNPT will be further improved by solving the problems mentioned above in the future.

## 6. Conclusions

This study mainly examined errors resulting from the deviation of each part of the hardware system, which were introduced by the deformation of the mechanical structure, the nonlinear characteristic of the electrical parts and the machining error of the mechanical parts. This study established the error function of the hardware system along the direction of the signal transmission and calculated the temperature measurement error caused by the deviation of the parameters in the hardware system using the system error function. Several significant factors (namely, the radius of the Helmholtz coils *r*_0_, the turns of the Helmholtz coils *N*_0_, the inductance of the Helmholtz coils *L*, the voltage gain of the power amplifier *k*_1_, the attenuation coefficient of the power amplifier within the pass band *k*_2_ and the signal source *U*_0_) were found to significantly affect the precision of the measured temperature. We also found that several errors counteracted one another in the process of analyzing the variation rule of the significant effect errors. Thus, the minimal error transfer module was proposed by the signal transmission direction, that is, the system error transfer module must follow Equation (15), which provided the reference for our design of the hardware system. Furthermore, the signal-to-AC bias ratio was expected to be higher than 80 dB, so that the AC bias of the system introduced inconspicuous errors in the final calculation.
